# Summary of Known Genetic and Epigenetic Modification Contributed to Hypertension

**DOI:** 10.1155/2023/5872362

**Published:** 2023-05-09

**Authors:** Tiar Masykuroh Pratamawati, Idrus Alwi, Asmarinah

**Affiliations:** ^1^Program Doctoral Biomedical Science, Faculty of Medicine, Universitas Indonesia, Jakarta, Indonesia; ^2^Department of Genetics, Faculty of Medicine, Universitas Swadaya Gunung Jati, Cirebon, Indonesia; ^3^Division of Cardiology, Department of Internal Medicine, Faculty of Medicine, Universitas Indonesia, Cipto Mangunkusumo National General Hospital, Jakarta, Indonesia; ^4^Department of Medical Biology, Faculty of Medicine, Universitas Indonesia, Jakarta, Indonesia

## Abstract

Hypertension is a multifactorial disease due to a complex interaction among genetic, epigenetic, and environmental factors. Characterized by raised blood pressure (BP), it is responsible for more than 7 million deaths per annum by acting as a leading preventable risk factor for cardiovascular disease. Reports suggest that genetic factors are estimated to be involved in approximately 30 to 50% of BP variation, and epigenetic marks are known to contribute to the initiation of the disease by influencing gene expression. Consequently, elucidating the genetic and epigenetic mediators associated with hypertension is essential for better discernment of its pathophysiology. By deciphering the unprecedented molecular hypertension basis, it could help to unravel an individual's inclination towards hypertension which eventually could result in an arrangement of potential strategies for prevention and therapy. In the present review, we discuss known genetic and epigenetic drivers that contributed to the hypertension development and summarize the novel variants that have currently been identified. The effect of these molecular alterations on endothelial function was also presented.

## 1. Introduction

Hypertension, defined by persistent elevated systolic blood pressure (SBP) and diastolic blood pressure (DBP) >140 mmHg and >90 mmHg, has remained to be a major global health challenge. 31.1% of the world's adult population with a prevalence of 28.5% in high-income countries (HICs) and 31.5% in low-/middle-income countries (LMICs) are estimated to have hypertension [1–3]. This proportion is known to have doubled from 1990–2019 and is expected to continue rising in the upcoming years [4]. Impairments in the integrated control systems of BP have been identified as the main driver of the disease [1, 5]. Nevertheless, hypertension pathophysiology is still concealed [6]. Whereas, identifiable causes such as parenchymal renal disease, aldosteronism, and renovascular hypertension are known to be involved in 5% of the cases, 90–95% of all hypertension cases are left undetermined [7, 8].

Studies found that the hypertension prevalence is diverse among ethnicities and families. Moreover, family history was discovered to be a nonmodifiable risk factor for the hypertension development demonstrated by a positive relationship of BP among siblings and between parents and offspring, suggesting a multifactorial nature of the disease [9–12]. Considering hypertension as a major risk factor for several lethal states of cardiovascular disease (CVD), such as heart attack, congestive heart failure, stroke, and peripheral vascular disease (PVD) [13], researchers have switched focus largely on searching for feasible molecular mechanisms, which may be associated with hypertension, and determining on how genetic variation may alter the BP architecture [14]. With the advancement in gene detection and mapping, a growing body of evidence has successfully demonstrated that hypertension results from a complex interaction among genetic, epigenetic, and environmental elements [15].

To date, approximately 30 to 50% of BP variation is known to be influenced by a genetic factor, and epigenetic modifications are evident to be associated with the hypertension development [7, 16–18]. Consequently, identifying genetic and epigenetic variants that are linked to the disease is important for resulting in a better discernment of the hypertension pathophysiology [7]. Scrutinizing epigenetics aspects could particularly elucidate processes that cannot be described by classic Mendelian inheritance [19]. Finally, elucidating the pathogenetic mechanisms that initiate hypertension development is essential to determine the potential effort required to prevent and cure the disease [20]. Unravelling its genetic basis could also be contributed to the knowledge of an individual's predisposition to hypertension and thus can accelerate the early enactment of preventive efforts and therapeutic plans [15].

The current review aims to provide an overview of known genetic and epigenetic modifications that contributed to hypertension development and summarize the currently identified novel variants. To the best of our knowledge, this is the first review to discuss both genetic and epigenetic factors associated with hypertension in conjunction.

## 2. Genetics of Hypertension

### 2.1. Monogenic Hypertension

Although it is widely accepted that hypertension is an outcome of complex multifactorial interaction, approximately 30% of the cases are inherited by a single genetic mutation which generally conforms to the Mendelian inheritance [21, 22]. This monogenic form is commonly associated with electrolyte disturbances and manifested as refractory hypertension, hypokalaemia, metabolic alkalosis, and low renin levels [23]. Also, the mechanisms that are recognized to explain the physiopathology of identified monogenic hypertension are excessive sodium ion reabsorption, excessive steroid synthesis, and excessive mineralocorticoid synthesis [24].

### 2.2. Apparent Mineralocorticoid Excess (AME)

AME is an ultrarare autosomal recessive disorder caused by a deficiency in an 11 beta-hydroxysteroid dehydrogenase type 2 (HSD11B2) enzyme due to the HSD11B2 gene mutation. Patients with AME display low birth weight, postnatal failure to thrive, severe juvenile low-renin, the onset of severe hypertension, and hypokalaemia [25]. Primarily expressed in sodium-transporting epithelia, the HSD11B2 enzyme facilitates the metabolic conversion of cortisol to its inactive form, cortisone, at aldosterone binding sites thus protecting mineralocorticoid receptors (MRs) from cortisol excess [25]. In the compromised HSD11B2 activity as a result of mutation, the MRs were overstimulated by cortisol causing intense water and sodium retention, hypokalaemia, and hypertension [26]. To date, about 40 causative mutations in the HSD11B2 gene have been recognized [27]. However, several novel mutations associated with AME have been identified.

Utilizing the next-generation sequencing (NGS) and Sanger sequencing, Fan et al. [26] found that a paternally inherited c.343_348del and maternally inherited c.1099_1101del variant were detected in exon 2 and 5 of a teenager with AME. Both mutations are characterized to result in Glu115 deletion, Leu116 deletion, a truncated 11*β*HSD2 protein production, and Phe367 deletion. Moreover, Bertulli et al. [28] reported that a novel homozygous frameshift variant in exon 5, c.900 dup, was found in a child with AME. In six affected patients from an Omani family, Yau et al. [27] revealed that a homozygous c.799A > G mutation within exon 4 of the HSD11B2 gene is causing NAD misalignment under p.T267A substitution. Meanwhile, Pizzolo et al. [29] and Alzahrani et al. [30] demonstrated that AME is associated with homozygous c.C662G variant and a missense biallelic mutation c.G526A of exon 3. The c.C662G mutation was identified to change alanine to glycine at position 221 leading to a chemical bond disturbance due to enzymatic affinity decline. Meanwhile, c.G526A mutation was evident to alter aspartic acid (GAT) to asparagine (AAT) at codon 176.

### 2.3. Liddle's Syndrome (LS)

LS, also known as pseudohyperaldosteronism, is a rare autosomal dominant inherited disorder clinically manifested by an early onset of hypertension, metabolic alkalosis, hypokalaemia, low renin levels, and suppressed aldosterone production [31, 32]. Genetic studies showed that this syndrome is caused by mutations in the terminal ends of the epithelial sodium channel (ENaC) [33]. ENaC is a membrane-bound ion channel primarily present in the apical portion of the aldosterone-sensitive epithelial cells such as the distal nephron, lung, and collecting duct. Composed of *α*, *β*, and *γ* homolog subunits containing a proline-tyrosine (PY) motif encoded by sodium channel epithelial 1 subunit alpha (SCNN1A), sodium channel epithelial 1 subunit beta (SCNN1B), and sodium channel epithelial 1 subunit gamma (SCNN1G) gene, respectively, this channel is responsible for Na^+^ reabsorption that provides the rate-limiting step for fluid intake into the bloodstream [34, 35]. PY motif (PPxY) is a conserved ubiquitination site of Nedd4 that mediates both internalization and degradation of ENaC. A point mutation in *β* or *γ* subunit of SCNN1B and SCNN1G genes has been characterized as the cause of the progression of the disease caused by a PY configuration change [32, 36].

Mutation in the PY motif is known to truncate the cytoplasmic COOH terminus of the ENaC *β* and *γ* subunits, impair the effective binding of Nedd4 to ENaC, and increase ENaC function that raises sodium reabsorption, elevate blood volume, and elevate BP [32, 37]. Since, the first nonsense p.Arg566^*∗*^ substitution reported by Liddle et al. in 1963, various causative mutations have been reported in recent years. In a total of thirteen individuals from a Czech family, Mareš et al. [38] reported that a novel nonsense mutation c.C1988A in the 13^th^ exon of the SCNN1B gene of ENaC *β* subunit was identified in 7 individuals causing a p.Tyr604^*∗*^ which induce a premature termination codon at amino acid position 604 shortening *β* subunit from 640 to 603 amino acids. Meanwhile, a frameshift mutation in exon 13 of SCNN1G, p.Arg586Valfs^*∗*^598, was identified by Fan et al. [39] resulting in a premature stop codon at the 598 positions and deleting the PY motif. Furthermore, Ding et al. [40] found that a new deletion c.1721delC of the SCNN1B gene was found to be associated with LS due to p.Pro574HisfsX675.

### 2.4. Glucocorticoid-Remediable Aldosteronism (GRA)

GRA is a rare hereditary cause of primary aldosteronism by which aldosterone is regulated by an adrenocorticotrophic hormone (ACTH) instead of the renin-angiotensin system. Patients with GRA are characterized by the clinical presentation of early-onset of severe hypertension with the low plasma renin activity (PRA) and mild hypernatremia in which synthetic glucocorticoid administration suppresses this mineralocorticoid excess [41, 42]. Identified as the most cause of monogenic hypertension, this is an autosomal dominant inherited trait due to a chimeric gene duplication arising from unequal crossing over between the 5′ adrenocorticotropin-responsive regulatory sequences of 11*β*-hydroxylase (CYP11B1) gene to the 3′ coding sequences of aldosterone synthase (CYP11B2) gene. The fusion is leading to ectopic expression of aldosterone synthase in the zona fasciculata of the adrenal cortex thereby aldosterone is released under ACTH [43, 44].

### 2.5. Gordon's Syndrome (GS)

GS, also known as type II pseudo-hypoaldosteronism or familial hyperkalaemia and hypertension, is an autosomal dominant inherited form of arterial hypertension characterized by elevated BP, hyperkalaemia, and metabolic acidosis. Individuals with GS usually show an effective efficacy of low-dose thiazide diuretics or sodium restriction suggesting that the loss of thiazide-sensitive NaCl cotransporter (NCCT) function in the distal convoluted tubules is involved in the initiation of the disease [45]. GS is interestingly distinguished from other syndromic forms by its normo- or hypokalaemia feature; thus, serum potassium is a useful determiner of the illness [46, 47]. Mutations in genes related to the regulation of the NaCl cosymporter NCCT, WNK1, WNK4, CUL3, and KLHL3 have later been discovered to be associated with GS and its varying severity [46].

Both WNK1 and WNK4 belong to WNK (with no lysine kinase) family that lacks a conserved lysine residue for ATP docking. Studies demonstrate that the wild-type WNK4 is a natural inhibitor of the thiazide-sensitive NCCT thus reducing membrane expression of NCCT and repressing the renal outer medullary potassium channel (ROMK). Missense mutations in WNK4 convert the action of WNK4 from NCCT and ROMK suppression leading to Na^+^ and K^+^ retention. Meanwhile, the biological function of WNK1 upon NCCT is still unclear. Nevertheless, WNK1 is known to contain WNK4-NCCT interaction thus deletion in WNK1 seems to be the gain-of-function due to increased expression [48–51]. On the other hand, Kelch-like 3 and Cullin 3 proteins encoded by CUL3 and KLHL3 genes assemble to form an E3 ubiquitin complex which in the basal state, interacts with the NCCT, ENaC, and ROMK as a regulator to control normokalaemia and normotension [47]. Reports suggest that the CUL3-KLHL3 E3 complex regulates BP through its ability to ubiquitylate WNK and that its connection with GS is due to the accumulation of WNK4 [52].

In addition to the known causative variant, various novel mutations have been identified associated with GS. Sakoh et al. [53] reported that a novel missense mutation of D564N in the acidic motif in WNK4 is identified in the mother and the proband of Japanese familial leads to the diagnosis of GS. The mutation is hypothesized to result in the WNK4 binding disruption leading to WNK4 protein excess. Moreover, Kelly et al. [54] found a missense mutation of c.1492C > T p.His498Tyr in the fifth kelch motif of the protein affecting exon 13 of the KLHL3. This finding supports the existing knowledge that the CUL3 and KLHL3 proteins are important regulators of the NCCT and thus are a potential target for a novel antihypertensive drug. The known mutations that contributed to monogenic hypertension are summarized in Table 1.

### 2.6. Polygenic Hypertension

#### 2.6.1. The Renin-Angiotensin-Aldosterone System (RAAS)

The renin-angiotensin-aldosterone system (RAAS) is a vital homeostatic regulator of arterial pressure, tissue perfusion, and extracellular volume by modulating blood volume, sodium reabsorption, potassium secretion, water reabsorption, and vascular tone. As the name indicates, renin and angiotensin are two critical components forming the system [55, 56]. Renin is produced in the specialized kidney granular cells called juxtaglomerular (JG) in its 406 amino acid-long precursor named prorenin. In response to the JG cell activation due to low arterial BP, low sodium chloride, or sympathetic nervous system activity, prorenin is converted to renin by which in the bloodstream, it acts on its target, angiotensinogen, and cleaves angiotensinogen into angiotensin I [57, 58]. In the vascular endothelium of the lungs and kidney, the decapeptide angiotensin I is further converted by the endothelial-bound angiotensin-converting enzyme (ACE) into an octapeptide hormone angiotensin II, a potent vasoconstrictor and the primary active product of the RAAS [55, 59].

The RAAS system is one of the well-elucidated systems upon its genetic predisposition to hypertension [7]. Among all the components, genetic polymorphism in angiotensinogen (AGT) and angiotensin-1-converting enzyme (ACE) are the most reliable candidate risk for hypertension given consistent findings. The candidacy of the AGT gene in hypertension was first described by Jeunemaitre and coworkers in Utah and Paris family. Through a sibship analysis, it is shown that AGT variants are associated with hypertension, and plasma concentrations of angiotensinogen were significantly different among hypertensive subjects with different AGT genotypes providing a possible mechanism for the genetic links [60]. The role of AGT on hypertension was strengthened by observations in animal models in which transgenic mice carrying overexpression of a rat angiotensinogen gene develop hypertension, while knockout mice with a disrupted corresponding gene product exhibit normal BP [61, 62]. Among all the identified polymorphisms of AGT, M235T, and T174M variants, showed the most significant association with hypertension in various in different populations [63, 64].

Although the contribution of the ACE gene to hypertension is still contradictory, consistent findings on the implication of insertion/deletion (I/D) polymorphism within intron 16 (II homozygote, ID heterozygote, and DD homozygote genotypes) of the ACE gene to the etiology of hypertension have been widely reported [65]. A case-control study among hypertensive patients and normotensive control groups found that the DD genotype and D allele of the ACE gene have had a strong association with a high risk of hypertension in the study population [66]. Furthermore, a recent meta-analysis study demonstrated that ACE I/D polymorphism is strongly associated with elevated BP in the dominant, recessive, and homozygous codominance models as well as in the allele contrast model. Individuals carrying the D allele were shown to have 1.49 times higher risk of the developing hypertension than patients with the I allele. In accordance, subjects with the DD genotype were at a 2.17 times greater risk of hypertension compared to the codominant genotype II, dominant (DD + ID vs. II), and recessive model [65]. Interestingly, some studies showed that genetic variants in ACE are gender-specific in that it influences BP more in males than in females [67, 68].

#### 2.6.2. G-Protein Coupled Receptor (GPCR) Kinases

G protein-coupled receptors (GPCRs) are the largest superfamily of integral membrane receptors that played as a key transducer in a variety of signal transduction systems for normal physiological processes. A variety of GPCRs are involved in the regulation of BP and the maintenance of normal cardiac function [69]. Upon stimulation, specific binding of G-protein to activated GPCR releases a rapid GDP ⟶ GTP nucleotide exchange and dissociates the GPCR-G-protein complex as well as the G-protein into free functional subunits, G*α* and G*βγ*, which stimulates the activation of downstream effectors. In the vasculature, GPCRs mediate BP by modulating the equilibrium between vasoconstriction and vasodilatation. Angiotensin II (Ang II) type 1 receptor (AT1R), *α*-adrenergic receptor, endothelin A receptor, and neuropeptide Y receptor are the vasoconstriction driver under GPCRs whereas acetylcholine receptors, *β*-adrenergic receptor, the endothelin B receptor, and the dopamine receptor are a GPCRs-induced mediator to promote vasodilation. The balance between these prohypertensive and antihypertensive shiftings is crucial to maintain normotensive states [70–72].

Recently, changes of cytosine to thymidine (C825T) in the *β*3 subunit of the G protein gene (GNB3) have been discovered in individuals with essential hypertension and considered as a candidate mutation for arterial hypertension. Zha et al. [73] reported that a decrease in the GRK2 (G-protein-coupled receptor kinase 2) ubiquitination levels, a protein vital for the GPCR desensitization process, and an increase in the GRK2 protein levels were identified in G*β*3 825T allele carriers resulting in the loss of the 41 amino-acid residues which disturb the G*β*3-DDB1 binding and alter the action of G*β*3s on GRK2 ubiquitination [73]. Moreover, El Din Hemimi et al. [74] demonstrated that the C825T allele is a risk factor for essential hypertension in the Egyptian population whereas a significant gender-specific effect of GNB3 C825T polymorphism on the serum sE-selectin levels was observed in males associated with CVDs outcomes [74, 75].

#### 2.6.3. Immune System and Inflammation

The implication of the immune response to hypertension genesis has been identified by a large number of investigations for decades. The initial study aimed to examine the role of immune cells in hypertension was done in the 1960s in rats with partial renal infarction. At the time, the investigators found that immunosuppression administration attenuates hypertension in the animal model and that transfer of lymphocytes from rats with renal infarction causes hypertension in normotensive recipient rats [76, 77]. To date, several immune mediators have been recognized for their possible role in hypertension initiation.

A study on subjects with pulmonary arterial hypertension (PAH) in China suggests that −572C/G promoter polymorphism in the interleukin-6 (IL-6) gene is associated with serum IL-6 levels and risk of hypertension [78]. Moreover, a meta-analysis showed that a −308G/A polymorphism in the tumor necrosis factor- *α* (TNF-*α*) increases the risk of essential hypertension in the Asian population [79]. Another meta-analysis involving 1,092 patients with essential hypertension and 1,152 controls found a significant association between the TNF*α* G308A gene polymorphism with hypertension [80]. Furthermore, a recent review indicated that there is a correlation between the serum TGF*β*1 levels, polymorphisms of the TGF*β*1 gene, and the severity of hypertension [81].  Table 2 are the summary of the known genetic variants that contributed to hypertension.

## 3. Epigenetic of Hypertension

Epigenetics is a heritable modification to the regulation of the gene activity, without changing the primary DNA sequence or genotype. In addition to genetic variants, epigenetic modifications have been demonstrated to play significant functions in the pathogenesis of hypertension [19].

### 3.1. DNA Methylation

DNA methylation is a stable and inheritable epigenetic modification to the control gene expression. It primarily occurs at cytosine in cytosine-guanine dinucleotides (CpG) islands involving the biochemical process of a methyl group (CH_3_) additionally derived from S-adenosyl-l-methionine to carbon number 5 of the pyrimidine ring in cytosine residue to form 5-methylcytosine (5mC). CpG islands are short sequences of palindromic DNA typically located within the promoter region or 5ʹ-end of a gene [17, 19]. In normal cells, CpG islands are mostly methylated, except for those located in the promoters, which are normally maintained unmethylated by unknown mechanisms [82]. Catalyzed by DNA–methyl transferases (DNMTs) enzyme, DNA methylation is a cellular mechanism used to suppress gene transcription by which hypermethylation results in gene silencing [83]. Several gene-specific DNA methylations have been reported related to hypertension.

### 3.2. AT1Ar and AT1b Methylation

Angiotensin II (Ang II) is a key effector of RAAS involved in BP and fluid volume control mediated by tissue-specific membrane receptors: angiotensin type 1 receptor (AT1R) and angiotensin type 2 receptor (AT2R). Expressed in multiple organs, the AT1R is composed of two functional subunits of the AT1a receptor (AT1aR) and AT1b receptor (AT1b). Activation of AT1R initiates the chronic hypertensive effect by the Ang II effect on the renal vasculature [84–86]. A study in male spontaneously hypertensive rats (SHRs) and age-matched Wistar–Kyoto rats (WKY) showed that the mRNA and protein expression of AT1aR was significantly higher in SHRs in comparison with WKY. A bisulfite sequencing analysis further demonstrated that the AT1aR promoter of the aorta and mesenteric artery of the SHRs was hypomethylated in contrast to the control rat suggesting that the AT1aR promoter hypomethylation might be responsible for the elevation of AT1aR expression in SHRs [86].

In accordance, in a study aimed to investigate the efficacy of losartan, a prehypertensive therapy, the mRNA and protein expression levels of AT1aR were significantly increased in high-fat-fed SHR rats, and out of seven promoter regions, five promoter areas were significantly hypomethylated [87]. On the other hand, a rat model of a maternal low-protein diet is utilized; Bodgarina and coworkers [88] have successfully demonstrated that in the first week of life, the expression of the AT1b receptor gene in the adrenal is overexpressed leading to upregulated receptor protein expression and enhanced angiotensin responsiveness. Pyrosequencing and *in vitro* analysis demonstrated that the proximal promoter of the AT1b gene in the adrenal is low methylated and that AT1b gene expression is strongly influenced by promoter methylation, respectively [88].

### 3.3. ACE Gene Methylation

Angiotensin-converting enzyme (ACE) is a key enzyme in RAAS that cleaves the C-terminal of the inactive angiotensin I (Ang 1) to the active Ang II [59]. A cross-sectional study in low-birth-weight children showed that there is a significant negative correlation between the ACE activity and BP with the level of DNA methylation [89]. Moreover, 24-h average exposure to fine particulate matter (PM) 2.5 among students in China was significantly associated with decreasing ACE methylation, increasing ACE protein, and increase SBP and DBP. ACE hypomethylation were shown to mediate the upregulation of ACE protein and elevation in ACE protein was found to associate with elevated BP [90]. In addition, a meta-analysis study found that overexpression of the ACE II gene due to DNA methylation results in vasoconstriction, increased peripheral resistance, and hypertension [91].

### 3.4. TLR4 and IL-6 Methylation

Toll-like receptor (TLR) is an important mediator of inflammatory pathways which play a major role in immune responses by recognizing a wide variety of pathogen-associated ligands derived from various microbe components [92]. Meanwhile, interleukin-6 (IL-6) is a member of the proinflammatory cytokine that plays a pivotal role in T-cell–mediated immunity and the proliferation and differentiation of nonimmune cells [93]. In accordance with hypertension, Mao et al. [94] demonstrated that hypomethylation in the CpG6 of the TLR2 promoter was significantly associated with the risk of hypertension [94]. Moreover, a cross-over trial of controlled-human exposure to concentrated ambient particles showed that reduced TLR4 methylation was associated with higher postexposure SBP and DBP [95]. Whereas, a significant correlation was discovered between the risks of essential hypertension with hypomethylations in the CpG site of IL-6 promoter in which the degree of DNA methylation was diverse among gender [96].

### 3.5. Histone Modification

Inside the cell, nuclear DNA is packaged into a highly condensed nucleosome with the help of two copies of histone proteins H2A, H2B, H3, and H4. Among all histone proteins, the N-terminal of H3 and H4 histone tails are subject to a variety of posttranslational modifications (PTM) that control chromatin structure and gene expression. Acetylation of nearby lysine residues, methylation of arginine residues, and phosphorylation of serine or threonine residues are the most occurring PTM product of the histone modification [17, 19]. In a salt-sensitive hypertension model of Wistar rats, Han et al. [97] demonstrated that upregulated histone H3K27me3 due to deoxycorticosterone acetate (DOCA) administration was significantly correlated with hypertension feature relief after resveratrol intake [97]. Moreover, a polymorphism in the DOT1L (rs2269879) and MLLT3 (rs12350051) genes were significantly associated with greater SBP and DBP which was possibly mediated by hypermethylation of H3 histone [98]. Meanwhile, Mu et al. found that a down-regulation in the WNK4 gene was shown to be associated with increased H3 and H4 acetylation, leading to upregulation of NCC which therefore promotes hypertension [99].

### 3.6. MicroRNA (miRNA)

miRNA is a single-stranded noncoding RNA that is involved in various cellular functions by serving as a posttranscriptional regulator of gene expression [100, 101]. It is transcribed in the nucleus from DNA sequences into primary miRNA (pri-miRNA), and then, it processed into a precursor miRNA (pre-miRNA) with the help of RNA polymerase II enzyme and a microprocessor complex consisting of polymerase III enzyme, Drosha, and the DiGeorge critical region 8 (DGCR8) protein [102]. After being exported to the cytoplasm by exportin 5 (XPO5)/RanGTP complex, pre-miRNA is cleaved by RNase III endonuclease resulting in ∼18- to 23-nucleotide-long mature miRNA duplexes [103]. It interrupts gene expression through the binding to the untranslated region of the messenger RNA (mRNA) resulting in protein synthesis alteration, mRNA destabilization, and gene expression repression. Since its first discovery, the correspondence of miRNA with hypertension has been widely reported [103–105].

Using a microarray, Li et al. [106] demonstrated that out of the total miRNAs detected in the experiment, 46 miRNAs were found to be profoundly expressed in hypertensive patients suggesting the miRNA contribution on the pathogenesis of hypertension [106]. Furthermore, dysregulation of miR-126-3p, 182-5p, and 30a-5p expression is evident to be the culprit of hypertension progression in the South African population meanwhile dysregulation of two novel miRNAs, miR-novel-chr1_36178 and miR-novel-chr15_18383, was significantly correlated with the SBP and DBP of the same population [104, 107]. In addition, an elevation of the circulated miR-505 plasma level is found among spontaneously hypertensive rats and angiotensin II-infused mice related to endothelial dysfunction and inflammation [105]. Understanding the role of miRNAs in hypertension is thus essential as it could add basic knowledge about the disease prognosis and possible therapeutic strategies.

## 4. Effect of Genetic and Epigenetic Alteration on Endothelial Function

The main untoward health consequence of hypertension is the increased risk for cardiovascular disease (CVD). It is estimated that about 54% of strokes and 47% of coronary heart diseases are attributable to elevated BP. Although the exact development cascade of hypertension to cardiovascular events remains to be elucidated, the occurrence of CVD is known to be associated with a compromised endothelial function [108, 109]. In the basal state, healthy endothelium is essential for the cardiovascular system as it is a key regulator of vascular tone through the release of endothelium-dependent relaxing factors such as nitric oxide (NO) and prostaglandins [110]. Moreover, it also exhibits antioxidant, anti-inflammatory, and antithrombotic properties that contribute to blood pressure control. In hypertension, the function of the vascular endothelium is altered characterized by attenuated endothelium-dependent vasodilatation and endothelial inflammatory activation which result in the initiation of the cardiovascular states [110, 111]. In accordance, modification in genetic and epigenetic architecture has been reported to result in endothelial dysfunction.

**Figure 1:**
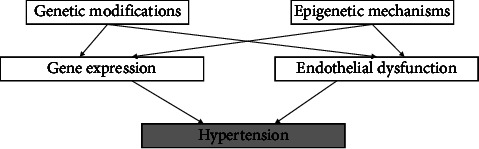
Genetic and epigenetic modification's role in hypertension. Hypertension can arise from genetic and epigenetic alteration which results in changes in gene expression and endothelial function.

Described in activatable Cul3∆9 and E-Cul3∆9 transgenic mice, it is reported that CUL3 mutation is associated with hypertension through reduced endothelial NO bioavailability, renovascular dysfunction, and increased salt-sensitivity of BP [112]. Furthermore, mice with 11*β*HSD2 knockout are shown to have endothelial dysfunction causing enhanced constriction to norepinephrine due to the impaired NO activity. Also, diminished relaxation responses to endothelium-dependent and -independent vasodilators and unaltered renal mineralocorticoid excess after fludrocortisone treatment were reported [113]. In terms of epigenetic basis, a growing body of evidence demonstrated that endothelial function is regulated by epigenetic mechanisms by which some genes need to be activated or silenced to achieve homeostasis. For instance, trimethylation of histone 3 lysine 27 (H3K27me3) is shown to contribute to endothelial gene expression alteration through gene silencing [114]. Moreover, histone acetyltransferase 7 (KAT7) appeared to involve in endothelial function by regulating the transcription of the vascular endothelial growth factors (VEGFs) [115]. Finally, the association between genetic and epigenetic modification with hypertension is summarized in Figure 1.

## 5. Conclusion

This current review has discussed the current known genetic and epigenetic modification contributed in the pathogenesis of hypertension derived from both the animal report and human experiments. The development in genetic and epigenetic basis of hypertension could result in the improved diagnosis, prognosis, and treatment of the disease.

## Figures and Tables

**Table 1:** A summary of novel genetic mutation associated with monogenic hypertension.

Diseases	Detection methods	Genes	Variants	Effects	Authors
AME	Next-generation sequencing, Sanger sequencing	HSD11B2	c.343_348del; c.1099_1101del	Glu115 and Leu116 deletion; Phe367 deletion	[26]
AME	Sanger sequencing	HSD11B2	c.900 dup	Glu301Argfs^*∗*^56	[28]
AME	Sanger sequencing	HSD11B2	c.799A > G	p.T267A substitution; misalignment of NAD in the coenzyme-binding site	[27]
AME	Pyrosequencing	HSD11B2	c.C662G	p.A221G substitution	[29]
AME	Sanger sequencing	HSD11B2	c.G526A	p.D176N substitution	[30]
LS	Next-generation sequencing, Sanger sequencing	SCNN1B	c.C1988A	p.Tyr604^*∗*^ substitution	[38]
LS	Sanger sequencing	SCNN1G	p.Arg586Valfs^*∗*^598	PY motif deletion	[39]
LS	Sanger sequencing	SCNN1B	c.1721delC	p.Pro574HisfsX675	[40]
GS	Sanger sequencing	*WNK4*	c.G1690A	p.D564N substitution	[53]
GS	Next-generation sequencing	*KLHL3*	c.1492C > T	p.His498Tyr substitution	[54]

The asterisk (^*∗*^) symbol represents a translation termination (stop) codon.

**Table 2:** A summary of genetic variant associated with polygenic hypertension.

Genes	Polymorphism(s)/markers
AGT gene	M235T and T174M variant
ACE gene	Insertion/deletion (I/D) polymorphism within exon 16, DD genotype, D allele
GNB3 gene	C825T allele
IL-6 gene	−572C/G promoter polymorphism
TNF-*α*	−308G/A polymorphism
TNF-*α*	G308A polymorphism

## Data Availability

The data used to support the findings of this study are available from the corresponding author upon reasonable request.
